# Socioeconomic Trends in Outcomes of Rehabilitation Among Patients With Systemic Connective Tissue Disorders in Germany: A Cross‐Sectional Analysis of Routine Data

**DOI:** 10.1111/1756-185X.70060

**Published:** 2025-01-10

**Authors:** Patrick Brzoska

**Affiliations:** ^1^ Health Services Research, Faculty of Health, School of Medicine Witten/Herdecke University Witten Germany

## Abstract

**Objective:**

Various demographic factors, including sex, socioeconomic status, and immigration status, have been linked to disparities in healthcare outcomes. Despite efforts by healthcare providers to address these inequities, interventions are not always effective. The present investigation provides empirical insights from Germany focusing on patients with systemic connective tissue disorders, highlighting the need for evaluated strategies to mitigate healthcare disparities.

**Methods:**

A 10% random sample of 2006–2016 routine data on patients with systemic connective tissue disorders (ICD‐10 M30–M36) is used. The sample included information on 1819 patients. The primary outcome assessed was the persistence of impairment following rehabilitation treatment. Logistic regression models were employed to adjust for demographic confounders. Interaction analyses were conducted to explore variations in disparities across different time periods and diagnostic groups.

**Results:**

Non‐German nationals were at 87% higher odds of impairment after treatment compared to German nationals (adjusted odds ratio [aOR] = 1.87; 95% confidence interval [CI] = 1.22–2.86). Furthermore, patients employed in semi‐skilled or unskilled positions demonstrated a 40% greater chance of poor outcomes compared to those in skilled occupations (aOR = 1.40; 95% CI = 1.03–1.90). Disparities in outcomes did not significantly vary across different years in which services were utilized.

**Conclusion:**

The study demonstrates disparities in healthcare outcomes associated with various diversity characteristics. These disparities are likely due to the different obstacles that some disadvantaged population groups encounter in the healthcare system. To address this heterogeneity, diversity‐sensitive healthcare provision strategies need to be implemented.

**Summary**

- The effectiveness of treatments aiming to mitigate the consequences of systemic connective tissue disorders may vary intersectionally across demographic and other categories.
- Using routine data, the study examines this intersectionality with respect to outcomes of rehabilitative treatments of patients with these conditions in Germany.
- The study identifies multiple disparities across demographic categories, highlighting the importance of an intersectional perspective in health research and practice.

## 1 Introduction

Systemic connective tissue disorders, such as systemic lupus erythematosus, systemic sclerosis, dermatomyositis, and polyarteritis nodosa, are a group of autoimmune conditions characterized by abnormal immune system activity affecting various organs and tissues, leading to a wide range of symptoms and complications. The management of systemic connective tissue disorders typically necessitates a multidisciplinary approach. This includes pharmacological interventions, physical therapy, and rehabilitative treatments that aim to enhance patients' functional abilities and quality of life [1]. Rehabilitation services, in addition to other preventive measures, are essential in reducing disability and enhancing the health‐related quality of life for individuals with these conditions. Such services are customized to address functional limitations, manage symptoms, and promote independence in daily activities. Rehabilitation services comprise physical and occupational therapy, psychological support, and other modalities to optimize overall well‐being and functionality. They focus on holistic care and personalized treatment approaches to mitigate disability progression and empower individuals to lead fulfilling lives despite the challenges posed by their conditions [2, 3]. In Germany, approximately 40 000 hospital cases of systemic connective tissue disorders are reported annually, and about 3000 individuals undergo rehabilitative treatment for these conditions every year [4].

Despite advances in medical treatment, individuals with systemic connective tissue disorders may face challenges in accessing appropriate care, especially if they belong to marginalized or disadvantaged groups [5, 6]. Healthcare disparities are a complex and multifaceted challenge that affects people worldwide, with demographic factors such as sex, socioeconomic status (SES), and migration status playing significant roles [7]. Despite extensive efforts to address health disparities, certain population groups continue to experience poorer health outcomes. International research shows that social determinants, for example, also significantly influence the outcomes for patients with systemic connective tissue disorders. Studies illustrate that individuals from lower socioeconomic backgrounds are more likely to experience delays in diagnosis, receive less comprehensive care, and have poorer health outcomes compared to those from higher socioeconomic backgrounds [8, 9, 10]. Migration status can also impact utilization of care, with immigrants and refugees often facing language barriers, cultural differences, and limited access to health services [11, 12]. Considering both their impact on the health of populations and their economic burden for the society [13], it is essential to understand the specific factors contributing to these disparities in different healthcare contexts to develop targeted and tailored interventions and policies that effectively address these inequities.

While some studies have been conducted on disparities in the utilization of healthcare services [14, 15, 16] as well as on disparities in general health outcomes [17, 18] in Germany, little insights are available on health outcomes for particular diagnostic groups. The objective of this study was to examine rehabilitative healthcare outcomes in Germany for patients with systemic connective tissue disorders. The study focuses on the association between demographic factors and post‐treatment impairment. By analyzing rehabilitative treatments over a 10‐year period, key determinants of health disparities within this population will be identified. Strategies for improving health equity will also be proposed. The findings of the study have the potential to impact clinical practice as well as healthcare policy and guide future research, helping to develop customized approaches to address healthcare disparities. By highlighting the key determinants of health disparities, the study will provide insights into how healthcare systems can be restructured to promote equity and efficiency, for example by informing the development of guidelines and best practices for the management of these conditions, ensuring that all patients receive high‐quality, comprehensive care regardless of their demographic background. Additionally, the results may reveal opportunities for higher efficiency in healthcare systems across Europe and other regions.

## 2 Methods

### 2.1 Data

For this investigation, a randomly selected 10% subset of data from the German Federal Pension Insurance regarding rehabilitation among working‐age individuals residing in Germany and undergoing rehabilitation due to systemic connective tissue disorders (ICD‐10 M30–M36) between 2006 and 2016 was utilized. The primary focus of the study was the assessment of the improvement of the health condition after rehabilitation (yes or no), evaluated at the point of discharge as part of a routine medical assessment [19].

### 2.2 Variables

Comparative analysis was performed across various demographic and socioeconomic variables such as age, sex, SES, and nationality groups (German national, non‐German national). SES proxies considered included employment type (full‐time, part‐time, unemployed, not applicable), occupation type (manual, service‐based, technical/professional, administrative, other), and occupational position (skilled labor, semi‐skilled/unskilled labor, trainee/unemployed). Additionally, the analysis was adjusted for marital status, treatment region, region of residence and health status represented by the duration of absence from work due to illness in the preceding 12 months.

### 2.3 Analysis

Multivariable logistic regression was used to explore factors associated with the improvement of health condition after rehabilitation, with adjusted odds ratios (aOR) and 95% confidence intervals (95% CI) calculated as effect estimates. Interaction analysis was conducted to explore variations in disparities over time and across diagnostic groups, considering changes in healthcare policies and advancements in treatment modalities over the study period [20]. Stata 16 was used for all analyses.

**TABLE 1:** Results of the multivariable logistic regression model with persistence of impairment after rehabilitation as the dependent variable (adjusted odds ratios [aOR] and 95% confidence intervals [95% CI]; 10% random sample of all individuals residing in Germany who underwent rehabilitation because of systemic connective tissue disorders [ICD‐10 M30–M36] during 2006–2016, *n* = 1,819; Main effects model. No interaction effects included; also adjusted for marital status, time absent from work before rehabilitation, year of service utilization).

	*N*	aOR	95% CI
Nationality			
German	1718	*Ref*	
Non‐German	101	1.87	1.22, 2.86
Sex			
Male	458	*Ref*	
Female	1361	1.01	0.77, 1.33
Age (in years)			
	1819	1.00	0.99, 1.01
Occupational position			
Skilled labor	1387	*Ref*	
Semi‐skilled/Unskilled labor	250	1.40	1.03, 1.90
Trainee/Unemployed	182	0.83	0.47, 1.48
Employment status			
Full time	1060	*Ref*	
Part‐time	442	1.22	0.94, 1.58
Unemployed	114	1.43	0.94, 2.18
Other	203	1.19	0.71, 2.01
Occupation			
Manufacturing	354	*Ref*	
Services	388	0.90	0.65, 1.25
Technical/Professional	367	0.79	0.55, 1.13
Administrative	395	0.86	0.61, 1.22
Other	315	0.74	0.51, 1.06
Diagnosis			
Polyarteritis nodosa and related conditions (M30)	43	*Ref*	
Other necrotizing vasculopathies (M31)	187	1.03	0.49, 2.18
Systemic lupus erythematosus (M32)	345	1.22	0.60, 2.50
Dermatopolymyositis (M33)	108	1.31	0.60, 2.86
Systemic sclerosis (M34)	193	1.33	0.63, 2.80
Other systemic involvement of connective tissue (M35)	932	1.23	0.62, 2.46
System. disorders of connective tissue in diseases classif. elsewhere (M36)	11	1.01	0.22, 4.61

*Note:* Ref.: Reference group. aO.

## 3 Results

The analysis included a total of 1819 cases with systemic connective tissue disorders, representing a diverse cohort with various demographic characteristics and clinical diagnoses. 74.7% of the patients were women. Among the included patients, 2.6% had polyarteritis nodosa and related conditions (M30), 10.4% had other necrotizing vasculopathies (M31), 19.0% had systemic lupus erythematosus (M32), 6.0% had dermatopolymyositis (M33), 10.4% had systemic sclerosis (M34), and 51.0% had other systemic involvement of connective tissue (M35). 32.4% of all cases were discharged from the hospital without any improvement in their medical condition according to the standardized medical evaluation.

**FIGURE 1:**
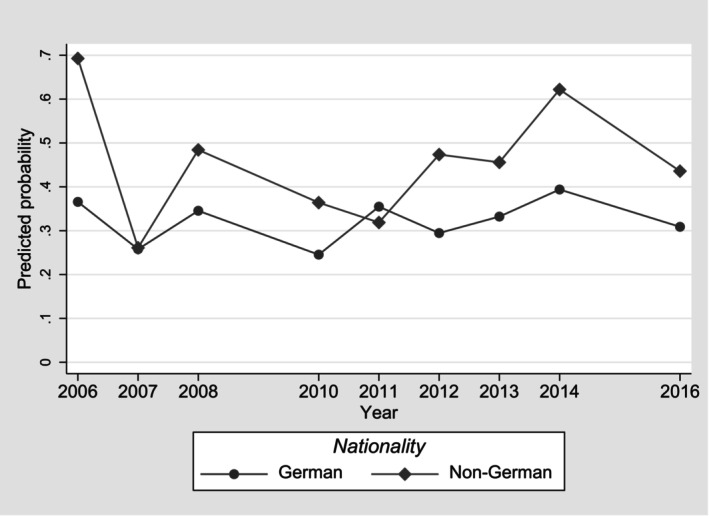
Predicted probability for persistence of impairment after rehabilitation by nationality and year of service utilization (10% random sample of all individuals residing in Germany who underwent rehabilitation because of systemic connective tissue disorders [ICD‐10 M30‐M36] during 2006–2016, *n* = 1819; Multivariable logistic regression model with interaction effects between nationality and year of service utilization).

The analysis revealed significant disparities in health outcomes among patients, particularly concerning demographic factors such as nationality and SES. Non‐German nationals exhibited a significantly higher likelihood of impairment post‐treatment compared to German nationals, with an aOR of 1.87 (95% CI = 1.22–2.86). Similarly, individuals employed in semi‐skilled or unskilled positions were at 40% higher odds of poor outcomes compared to those in skilled occupational positions, with an aOR of 1.40 (95% CI = 1.03–1.90) (Table 1). Disparities varied between the years in which services were utilized (in Figure 1 exemplarily shown for disparities between German and non‐German nationals); however, differences were not statistically significant. There were also no significant difference between men and women, between different age or between diagnostic groups.

## 4 Discussion

The findings of this study underscore the significant impact of demographic factors on health outcomes among patients with systemic connective tissue disorders in Germany. Non‐German nationals and individuals in lower SES occupations were identified as particularly vulnerable populations, highlighting the need for targeted interventions to address these disparities. The findings align with previous research demonstrating the influence of social determinants on disease outcomes and healthcare utilization patterns [21, 22].

Several potential mechanisms may contribute to the observed disparities in health outcomes among patients. Access to care, including barriers related to language, may play a significant role, particularly for non‐German nationals. Additionally, cultural factors and health beliefs may influence patients' adherence to treatment regimens and engagement with rehabilitative services [16]. Provider bias and disparities in quality of care may also contribute to differences in health outcomes, particularly for patients from marginalized or disadvantaged backgrounds [23]. To address these disparities effectively, targeted interventions and policies are needed to improve access to high‐quality care for all patients, regardless of their demographic characteristics. These must include increasing awareness and education among healthcare providers about the specific needs of diverse patient populations, as well as advocating for systemic changes in healthcare delivery to promote equity. Particularly, diversity‐sensitive measures in healthcare provision, including culturally competent care, language interpretation services, and outreach programs targeting underserved communities, can help mitigate barriers to care and improve health outcomes among vulnerable populations. Using an intersectionality framework helps stakeholders understand the needs and perspectives of healthcare users better, leading to more inclusive and effective healthcare outcomes. This approach promotes patient‐centeredness within healthcare services [24]. Although hospitals and rehabilitation centers recognize the need for culturally sensitive care, they still struggle to adopt appropriate strategies to promote it [25]. This is mainly due to the lack of accessible and comprehensive resources that offer practical guidance. Considering this limitation, in Germany, a manual providing tools and recommendations for implementation has been developed to address the gap in culturally sensitive healthcare practices within rehabilitation facilities [26]. The type of measures implemented depends on the specific characteristics of the facilities involved, its structures, and goals, which must be identified through a needs assessment. Key instruments at the disposal of facilities include diversity‐sensitive mission statements, ethical standards, and workplace agreements aimed at addressing discrimination and valuing diversity as an asset. Additionally, different forms of diversity trainings can enhance the awareness among staff for cultural and demographic differences, foster their self‐reflection, and promote cross‐cultural skills. Diversity‐sensitive HR policies can eliminate bias and promote team diversity. Tailored measures address specific needs, such as accessible language, equal treatment policies, inclusive spaces, and cultural mediators for patients and staff. Furthermore, efforts to address social determinants of health, such as poverty, education, and housing, are essential for achieving health equity and reducing disparities in chronic disease outcomes [27].

The strength of the present investigation is its ability to draw from a representative routine data source spanning a decade. This offers a unique opportunity to explore trends and patterns in health outcomes among individuals with connective tissue disorders undergoing rehabilitation. It is also important to take several limitations into account. Individuals may have had varying health statuses prior to rehabilitation, which could affect their outcomes regardless of the intervention. Using sickness‐related absences from work as the sole measure of health status may introduce residual confounding. This is even more so the case as individuals who were not engaged in paid work prior to rehabilitation were included in a separate category of the control variable “time absent from work before rehabilitation” (“other/not applicable”). This category, therefore, includes individuals with different health statuses.

Although the outcome measure “improvement of the health condition after rehabilitation” was based on a medical evaluation at the time of completion of treatment, it is limited by the fact that it is not based on a pre‐post comparison of the health condition but rather represents the treating physician's evaluation at the time of the patient's discharge from rehabilitation. Consequently, an overestimation of treatment success due to information bias is possible.

To improve the robustness of the examination, future research should consider incorporating additional data sources and variables. It is recommended to collect information on patients' initial health status, functional abilities, and quality of life measures before rehabilitation to provide a more comprehensive understanding of their baseline characteristics. Similarly, collecting data on patients' attitudes towards treatment, expectations, and preferences can provide valuable insights into the factors that influence their outcomes. Providing detailed information on the rehabilitation process, including the type and intensity of therapies, treatment duration, and adherence to prescribed interventions, would enable researchers to evaluate the impact of specific components of rehabilitation on health outcomes. Efforts to extend routine data with supplementary sources, such as patient‐reported outcomes, other electronic health records, survey data, or qualitative interviews are necessary to fill gaps in information. This will provide a more nuanced understanding of the complex factors that influence health outcomes in individuals undergoing rehabilitation for connective tissue disorders and other chronic diseases. The present study is also limited regarding the identification of immigrant status using nationality as a proxy. This method fails to fully capture the heterogeneity within the immigrant population. Consequently, immigrants with German citizenship may be inaccurately grouped with individuals of German nationality, potentially masking important differences in health outcomes and access to healthcare services within this population. Similarly, no information on ethnicity, place of birth or the length of stay in Germany was available. Addressing these limitations is crucial to ensure the validity and generalizability of study findings, especially in diverse societies where healthcare disparities may be influenced by factors such as immigration status, language barriers, and cultural beliefs.

## 5 Conclusion

The study provides empirical evidence of significant disparities in healthcare outcomes among patients with systemic connective tissue disorders in Germany. Non‐German nationals and individuals in lower SES occupations were identified as particularly vulnerable populations, highlighting the need for targeted interventions and policies to address these inequities. By implementing diversity‐sensitive measures in healthcare provision and addressing social determinants of health, policymakers and healthcare providers can work towards achieving greater health equity for all individuals, regardless of their demographic characteristics. Continued research and advocacy efforts are essential to ensure that all patients receive the care and support they need to achieve optimal health outcomes.

Future research in this area should focus on further elucidating the mechanisms underlying disparities in health outcomes among patients with systemic connective tissue disorders, including the role of access to care, provider bias, and social determinants. Longitudinal studies are needed to assess the long‐term impact of targeted interventions and policies on reducing health disparities within this population.

## Conflicts of Interest

The author declares no conflicts of interest.

## Ethics Statement

The study uses routine data which fully complies with Germany's Federal data protection act and the requirements of the German Social Code. This data is anonymous and did not involve any experiments, which is why no further ethical approval was required in accordance with national standards for routine data analyses in Germany.

## Data Availability

Qualified researchers who meet the criteria for accessing confidential data can obtain the datasets underpinning this study's findings at no cost from the German Federal Pension Insurance. Additional details are available at: https://www.eservice‐drv.de/FdzPortalWeb/dispcontent.do?id=main_fdz_english.

## References

[1] J. Y. Streifler and Y. Molad , “Connective Tissue Disorders: Systemic Lupus Erythematosus, Sjögren's Syndrome, and Scleroderma,” Handbook of Clinical Neurology 119 (2014): 463–473. 24365313 10.1016/B978-0-7020-4086-3.00030-8

[2] N. Alpiner , T. H. Oh , S. R. Hinderer , and V. A. Brander , “Rehabilitation in Joint and Connective Tissue Diseases. 1. Systemic Diseases,” Archives of Physical Medicine and Rehabilitation 76 (1995): S32–S40. 7741628 10.1016/s0003-9993(95)80597-4

[3] S. M. Bongi , A. Del Rosso , F. Galluccio , et al., “Efficacy of a Tailored Rehabilitation Program for Systemic Sclerosis,” Clinical and Experimental Rheumatology 27 (2009): S44. 19796561

[4] Federal Statistical Office , Hospital Statistics ‐ Diagnostic Data of the Hospital Patients (Fachserie 12 Reihe 6.2.1) (Wiesbaden: Federal Statistical Office/Statistisches Bundesamt, 2022).

[5] B. Hasan , A. Fike , and S. Hasni , “Health Disparities in Systemic Lupus Erythematosus–A Narrative Review,” Clinical Rheumatology 41 (2022): 3299–3311. 35907971 10.1007/s10067-022-06268-y PMC9340727

[6] D. F. Moore and V. D. Steen , “Racial Disparities in Systemic Sclerosis,” Rheumatic Disease Clinics 46 (2020): 705–712. 32981647 10.1016/j.rdc.2020.07.009

[7] M. Marmot , “Social Determinants of Health Inequalities,” Lancet 365 (2005): 1099–1104. 15781105 10.1016/S0140-6736(05)71146-6

[8] L. A. González , S. M. A. Toloza , G. McGwin, Jr. , and G. S. Alarcón , “Ethnicity in Systemic Lupus Erythematosus (SLE): Its Influence on Susceptibility and Outcomes,” Lupus 22 (2013): 1214–1224. 24097993 10.1177/0961203313502571

[9] E. W. Karlson , L. H. Daltroy , R. A. Lew , et al., “The Relationship of Socioeconomic Status, Race, and Modifiable Risk Factors to Outcomes in Patients With Systemic Lupus Erythematosus,” Arthritis & Rheumatism: Official Journal of the American College of Rheumatology 40 (1997): 47–56. 10.1002/art.1780400108 9008599

[10] K. Phillippi , M. Hoeltzel , A. B. Robinson , et al., “Race, Income, and Disease Outcomes in Juvenile Dermatomyositis,” Journal of Pediatrics 184 (2017): 38–44. 28410093 10.1016/j.jpeds.2017.01.046 PMC5410644

[11] M. Piram , C. Maldini , and A. Mahr , “Effect of Race/Ethnicity on Risk, Presentation and Course of Connective Tissue Diseases and Primary Systemic Vasculitides,” Current Opinion in Rheumatology 24 (2012): 193–200. 22249352 10.1097/BOR.0b013e32835059e5

[12] A. G. Uribe and G. S. Alarcón , “Ethnic Disparities in Patients With Systemic Lupus Erythematosus,” Current Rheumatology Reports 5 (2003): 364–369. 12967518 10.1007/s11926-003-0022-8

[13] T. A. LaVeist , D. Gaskin , and P. Richard , “Estimating the Economic Burden of Racial Health Inequalities in the United States,” International Journal of Health Services 41 (2011): 231–238. 21563622 10.2190/HS.41.2.c

[14] D. Wahidie , Y. Yilmaz‐Aslan , and P. Brzoska , “Participation in Colorectal Cancer Screening Among Migrants and Non‐Migrants in Germany: Results of a Population Survey,” Gastrointestinal Disorders 4 (2022): 97–107.

[15] C. Wiessner , T. Keil , L. Krist , et al., “Personen Mit Migrationshintergrund in der NAKO Gesundheitsstudie–Soziodemografische Merkmale und Vergleiche Mit der Autochthonen Deutschen Bevölkerung,” Bundesgesundheitsblatt, Gesundheitsforschung, Gesundheitsschutz 63 (2020): 279–289. 32034443 10.1007/s00103-020-03097-9

[16] J. Klein and O. dem Knesebeck , “Inequalities in Health Care Utilization Among Migrants and Non‐Migrants in Germany: A Systematic Review,” International Journal for Equity in Health 17 (2018): 1–10. 30382861 10.1186/s12939-018-0876-z PMC6211605

[17] O. dem Knesebeck , D. Lüdecke , and J. Klein , “Social Disparities in Access and Quality of Consultation in Outpatient Care in Germany,” BMC Primary Care 25 (2024): 299. 39143514 10.1186/s12875-024-02552-9 PMC11323346

[18] P. Brzoska , “Disparities in Health Care Outcomes Between Immigrants and the Majority Population in Germany: A Trend Analysis, 2006‐2014,” PLoS One 13 (2018): e0191732. 29360874 10.1371/journal.pone.0191732 PMC5779703

[19] Deutsche Rentenversicherung Bund , Der ärztliche Reha‐Entlassungsbericht. Leitfaden zum einheitlichen Entlassungsbericht in der medizinischen Rehabilitation der gesetzlichen Rentenversicherung 2007 (Berlin: Deutsche Rentenversicherung Bund, 2007).

[20] B. G. Tabachnick and L. S. Fidell , Using Multivariate Statistics (Boston: Pearson, 2012).

[21] L. E. Flores , M. Verduzco‐Gutierrez , D. Molinares , and J. K. Silver , “Disparities in Health Care for Hispanic Patients in Physical Medicine and Rehabilitation in the United States: A Narrative Review,” American Journal of Physical Medicine & Rehabilitation 99 (2020): 338–347. 31688009 10.1097/PHM.0000000000001342

[22] L. R. Castellanos , O. Viramontes , N. K. Bains , and I. A. Zepeda , “Disparities in Cardiac Rehabilitation Among Individuals From Racial and Ethnic Groups and Rural Communities–A Systematic Review,” Journal of Racial and Ethnic Health Disparities 6 (2019): 1–11. 29536369 10.1007/s40615-018-0478-x

[23] W. J. Hall , M. V. Chapman , K. M. Lee , et al., “Implicit Racial/Ethnic Bias Among Health Care Professionals and Its Influence on Health Care Outcomes: A Systematic Review,” American Journal of Public Health 105 (2015): e60–e76. 10.2105/AJPH.2015.302903 PMC4638275 26469668

[24] C. M. Tucker , T. M. Arthur , J. Roncoroni , W. Wall , and J. Sanchez , “Patient‐Centered, Culturally Sensitive Health Care,” American Journal of Lifestyle Medicine 9 (2015): 63–77.

[25] F. Erdsiek , T. Aksakal , M. Mader , et al., “Diversity‐Sensitive Measures in German Hospitals–Attitudes, Implementation, and Barriers According to Administration Managers,” BMC Health Services Research 22 (2022): 1–9. 35606740 10.1186/s12913-022-08058-3 PMC9128136

[26] T. Aksakal , M. Mader , K. Annac , et al., “Unterstützung von Rehabilitationseinrichtungen bei der Umsetzung einer diversitätssensiblen Versorgung,” Entwicklung der DiversityKAT‐Handreichung. Die Rehabilitation 63 (2024): 23–30. 37722412 10.1055/a-2138-9199

[27] A. F. Brown , G. X. Ma , J. Miranda , et al., “Structural Interventions to Reduce and Eliminate Health Disparities,” American Journal of Public Health 109 (2019): S72–S78. 30699019 10.2105/AJPH.2018.304844 PMC6356131

